# Changes during the sleep onset process on EEG activity and the components of attention

**DOI:** 10.5935/1984-0063.20210010

**Published:** 2022

**Authors:** Jorge Borrani, Alán Chapa-Guerra, Vania De-la-Garza, Iván López, Alba Isaias, Luisa Pimentel-Rodríguez, Aída García, Candelaria Ramírez, Pablo Valdez

**Affiliations:** Universidad Autónoma de Nuevo León, School of Psychology, Laboratory of Psychophysiology - Monterrey - Nuevo León - Mexico.

**Keywords:** Attention, Sleep, Electroencephalography, Spectral Analysis, Sleep Onset, Continuous Performance Task

## Abstract

The sleep onset process (SOP) happens every time a person falls asleep, regardless of the time of day or if they are doing an activity. Basic cognitive processes, such as attention, differ between wakefulness and sleep. The components of attention - tonic alertness, phasic alertness, selective, and sustained attention - are known to decrease during sleep, however they have not been analyzed during the sleep onset process. This study analyses the state of three of the four components of attention during the sleep onset process through electroencephalographic (EEG) activity and task performance in young people. Nine undergraduate students (18.54±1.24 years old) underwent a control session which was compared to the average of four sleep-inducing sessions. During all sessions, the EEG activity of the subjects was recorded to assess the effect of the SOP on electroencephalographic activity while they answered a continuous performance task (CPT) to assess the effect of the SOP on the components of attention. Comparisons of the EEG recordings of the control and the sleep inducing sessions demonstrated that there is lower activity in fast beta, as well as a higher theta and delta activity right before the sleep onset. There was a decrease in tonic alertness, phasic alertness, and selective attention. This study shows that there is an increase in EEG slow activity and a decrease in fast activity, as well as in attentional capacity during the SOP. This decrease can become a safety hazard since it could happen while performing daily activities.

## INTRODUCTION

The sleep onset process (SOP) is the transition from wakefulness to sleep. The SOP consists of a series of states with cognitive and physiological characteristics that differ from those of sleep and wakefulness^1^. The SOP occurs every time a person is falling asleep. It happens during the night at bedtime, but it may occur at any time of the day, even while performing tasks or activities^2,3^.

The propensity to enter the sleep onset process during the day increases with sleep deprivation^4^. It is considered that 70% of college students do not get sufficient sleep^5^, which implies that most young people are at risk of entering the SOP while performing an activity, this performance depends on cognitive processes such as attention. Understanding cognitive changes during the SOP would help prevent accidents in young people that are sleep deprived, but in order to do so, it is important to analyze both the electroencephalographic (EEG) and cognitive changes during the SOP.

The EEG signal during the SOP has been visually described as an appearance and disappearance of alpha waves, followed by theta waves, vertex sharp waves, and ending with the K complexes and sleep spindles of stage 2 of the Rechtschaffen & Kales scoring system^6^. Performing a visual analysis only takes into account the dominant frequency of a signal, but in order to detect more specific and subtle changes it is necessary to perform a spectral analysis.

Having more activity in a faster frequency band indicates that more neurons in the area under the electrode are firing at a fast and desynchronized rate. EEG fast frequencies include gamma, beta, and alpha activity. Beta activity is associated with wakefulness, and it increases during attention tasks, particularly in the right hemisphere of the brain^7,8^. Nevertheless, these relations have only been observed by dividing the beta band into sub-bands^9^. The presence of alpha indicates that a person is still awake, but that the sensory stimuli processing capacity is weakening^10,11^. Delta and theta are considered slow frequencies, which indicate a lower level of alertness and an impaired ability to process and respond to stimuli^12,13^.

Right before falling asleep there is a decrease in fast frequencies and an increase in slow ones^14^, which suggests that in this moment there could be a decrease in cognitive capacity^15,16^. Nevertheless, there are no studies that analyze the EEG signal in relation to cognitive performance during the SOP, specifically the decrease in responses to the environment.

Responding to the environment depends on attention, which is a basic cognitive process necessary to perform most daily activities. It consists of four components, but in this study, only the following three will be analyzed: tonic alertness, the general capacity to respond to the environment; phasic alertness, the capacity to respond to stimuli after a warning signal; and selective attention, the capacity to give specific responses to specific stimuli^17,18^. These components of attention are related to specific brain circuits. Tonic alertness is related to the inferior nuclei of the reticular activating system, and phasic alertness is related to the superior nuclei of the reticular system, particularly the cerebral colliculus. Selective attention is related to the prefrontal cortex and its connections to the posterior parietal cortex. Sustained attention is related to the influences of the prefrontal cortex on the reticular activating system^19^.

There are no studies that directly assess all components of attention during the SOP, but there is some indirect evidence through studies that evaluated performance during this process. One of these studies employed an auditory task in which participants had to press a button when they heard a sound, which can be interpreted as a measure of tonic alertness. This study compared sleep latencies in college students between 21 and 26 years of age using a behavioral criterion (absence of responses) versus polysomnographic sleep onset criteria. The results showed a longer latency when using the polysomnographic criteria compared to the behavioral criterion, which means that participants first stop responding and, after a while, the characteristic events of sleep stage 2 such as K complexes appear^20^. Even though task performance was not reported, these results suggest that people stop responding because their attention decreases, not because of the muscle tone and sensory decrease typical of sleep stage 2. Another study in young adults between 25 and 35 years of age showed, with a similar auditory task, that reaction time increased from 700 milliseconds during alpha activity to almost two seconds during the appearance of sleep spindles and K complexes^21^. These results are also evidence that as the sleep onset approaches, the capacity of these young people to respond in general deteriorates. Nevertheless, since these tasks only have indices for tonic alertness, the other components of attention are yet to be analyzed.

It is important to analyze attention through a single task that has indices for all components, such as a continuous performance task (CPT)^22,23^. Through this task, it is possible to analyze all components of attention at the same time and detect a decrease in a specific component, which would suggest the types of activities that could be more affected during the SOP. An auditory CPT would cause minimum disruption or delay in the SOP^24^, making it possible to test the hypothesis that the components of attention decrease during the sleep onset process.

In summary, the evidence suggests that during the SOP, attention decreases and there is a general synchronization of the EEG signal, but it is yet unclear what the state of the components of attention is during this period. A decrease in the components of attention while lying in bed at night is expected and poses no risk, but if this decrease happens during the day while performing an activity, it could lead to severe accidents. Therefore, the objective of this study is to analyze EEG activity and the components of attention during the sleep onset process.

## MATERIAL AND METHODS

### Participants

Nine undergraduate students of 18.54±1.24 (mean ± standard deviation) years of age, 6 females and 3 males, participated voluntarily. None of the participants reported sleep disorders or other illnesses, nor having traveled across more than two time zones during the previous three months. Participants signed a written consent form before cooperating in the study; those who were underage were allowed by their parents to participate by signing a similar form. An academic committee of the university approved this study and it was carried out according to the ethical standards of the declaration of Helsinki.

### Questionnaires

To ensure participants did not have sleep disorders or other illnesses and to record their sleeping habits, they answered three questionnaires:

General information questionnaire: a brief set of questions that gather demographic data, class schedules, and information on physical health and medication use;

Sleep disorders questionnaire: the self-report questionnaire was used to identify symptoms of sleep disorders such as insomnia, hypersomnia, and parasomnias;

Sleep diary: a daily log in which participants report bedtime and waking time.

### Polysomnographic recordings

Polysomnographic recordings were carried out using a Grass Comet AS40 EEG/PSG Amplifier System, using nineteen scalp electrodes placed according to the international 10/20 system. Ear lobe electrodes (A1 and A2) were bridged to REF to act as a reference electrode and an electrode on the forehead was used as the body ground (GND). These data were stored in a computer for offline analysis.

### Auditory continuous performance task (CPT)

To assess the components of attention during the sleep onset process, participants responded to an auditory continuous performance task. The auditory CPT is a modified version of the visual continuous performance task^18^ but with auditory stimuli that allow the assessment of the different components of attention. The participants were instructed to use their dominant hand to press key number 1 when a high-pitched sound (1900Hz) appeared, to press key number 2 to a medium-pitched sound (1400Hz), and to press key number 3 if a low-pitched sound (1000Hz) took place after a medium one. Stimulus duration is 100ms, and the inter-stimulus interval varies randomly ∼1200ms (1000, 1100, 1200, 1300, and 1400ms). The total duration of the task is 11.70min and it is divided into 27 blocks that consist of 14 high tones, 4 medium tones, and 2 low tones that follow the medium tone. Stimuli within the blocks are randomized in each application. According to the definition of each component of attention^17,18^ responses to the high tone were taken as indices of tonic alertness, responses to the mid-tone were taken as indices of selective attention, and responses to the low tone after the mid-tone were taken as indices of phasic alertness.

### Procedure

Participants signed a written informed consent letter and then answered the questionnaires. Only those without sleep disorders were included in the study. They kept a sleep diary for two weeks and those without a regular sleep cycle were not included in the study. All participants were asked to abstain from consuming alcohol, caffeine, energetic beverages, or any other drug, two days before and during all recording sessions. All nine participants went through five recording sessions in the laboratory: a control session in which they were not sleep deprived and four sleep-inducing sessions after a night of sleep deprivation - therefore, participants included in the control session are the same as those included in the sleep-inducing sessions. The protocol during these sessions is similar to a maintenance of wakefulness test^25^; in this case, participants were encouraged to stay awake and respond to the auditory CPT, in order to analyze the components of attention during the sleep onset process. They trained for the CPT the day before the control session.

**Figure 1:**
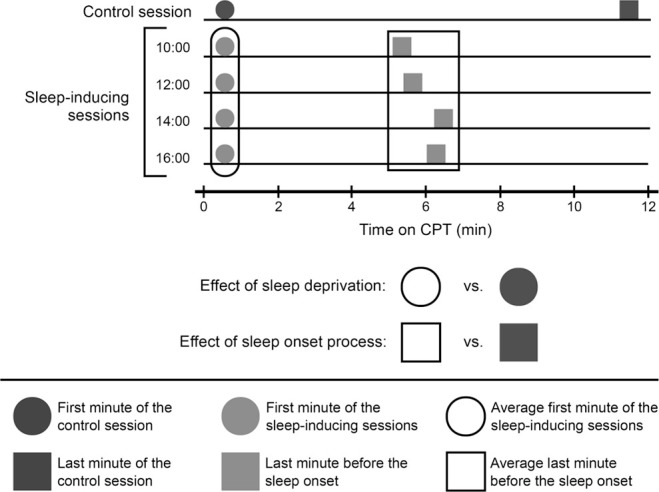
Schematic diagram of the moments analyzed in the study. EEG and performance data on the last minute of the task in the control session was compared to the averaged responses on the last minute before the sleep onset in the sleep-inducing sessions to analyze the effect of the SOP on EEG activity and the components of attention. EEG and performance data on the first minute of the task in the control session was compared to the averaged responses on the last minute before the sleep onset in the sleep-inducing sessions to analyze the effect of sleep deprivation on EEG activity and the components of attention.

The night before the control session, participants were asked to sleep freely (without setting an alarm clock) to promote a night of satisfactory sleep. The control session began between 12:00 and 14:00h. The night before the sleep-inducing sessions, participants were asked to sleep less than 7h, to promote sleep pressure. For the sleep-inducing sessions, participants arrived at the laboratory at 19:30h and remained awake all night. Trained collaborators ensured that the participants stayed awake by always staying with them, either talking, playing video games, or board games. Electrode placement started the next day at 08:00h and the recording sessions were scheduled at 10:00, 12:00, 14:00, and 16:00h (Figure 1).

All participants were recorded individually while laying on a bed, in a quiet isolated room with controlled room temperature at 24±1°C, which became completely dark when the lights were turned off (light level <1 lux). EEG activity was recorded while participants responded to the CPT. Infrared live video and polysomnographic recordings were monitored from a separate room. At the end of this period, the lights were turned on, participants were awakened (if necessary), and were allowed to leave the recording room. During the periods in between recordings, participants were accompanied at all times to verify that they remained awake and kept a low activity level. Two meal options around 400 calories were offered to participants at 9:00h and 13:00h.

### Data analysis

To analyze the SOP, it is crucial to determine first the sleep onset. Although there is no agreement on which is the precise point for the sleep onset^24^, in this study, the sleep onset was determined as the beginning of the first 30-second epoch of uninterrupted sleep stage 2^11,26,27^. These criteria were chosen in order to allow the observation of task performance up to sleep stage 2, when sensory perception and muscular tone reduce importantly. The C3, C4, O1, O2, Fz, and Cz channels were used for the visual analysis of the EEG recording.

Four channels were selected for spectral analysis: C3 and C4 because many sleep phenomena are observable in these channels, as well as Fp1 and Fp2 because these prefrontal areas are related to selective attention, Fz was included to have a vertex region that could show subcortical activity^28^. EEG recording files were converted into EDF for analysis. The unfiltered EEG signal was analyzed through a fast Fourier transform (FFT; Spike 2 version 6.18 script, SUDSA, version 2.2) in order to obtain a power spectrum in logarithmic scale (dB). Power spectrum was analyzed on 60s of performance during the CPT divided into non-overlapping 2s epochs (sampling frequency 128Hz), epochs with visual noise or extreme power spectrum values were discarded manually. Power was analyzed in each of the following frequency bands: delta (1-3Hz), theta (4-7Hz), alpha 1 (8-9Hz), alpha 2 (10-12Hz), beta 1 (17-20Hz), beta 2 (21-26Hz), and beta 3 (27-34Hz).

Performance during the last two blocks of the CPT before the sleep onset was the indicator for changes in the components of attention during the SOP. The duration of each block is approximately 28 seconds, so the performance analyzed was about 56 seconds long and included 40 stimuli. Spectral power during these same blocks was the indicator for changes in EEG activity during the SOP.

Since sleep deprivation was used to induce the SOP, it is necessary to dissociate the changes due to the SOP from the changes due to sleep deprivation. In order to achieve this, indicators for the components of attention and EEG activity were compared between the sleep-inducing sessions and the control session. Since there was no sleep onset during the control session, the selected blocks for comparison were the last two of CPT, since participants were under the same low-stimulation conditions as the sleep inducing session and similar fatigue from performing the CPT for almost 12 minutes^29^ (Figure 1). If during the sleep-inducing sessions, there is less spectral power on fast EEG bands and less correct responses compared to the control session, it would mean that the approaching of the sleep onset reduces the capacity to respond.

CPT performance and spectral power during the first two blocks of the task were the indices for changes in the components of attention and EEG activity due to sleep deprivation (Figure 1). At this moment, the SOP has not advanced much, and all participants were awake and responding, so the only difference between the control session and the sleep inducing sessions was the sleep deprivation of the sleep-inducing sessions. If at the beginning of the task there are differences in spectral power and correct responses, these would be due to sleep deprivation and not due to sleep onset.

The midday increase in sleep propensity^2^ was analyzed by comparing the individual performance on the CPT from the four sleep-inducing sessions through Friedman’s ANOVA. Since there were no differences with the time of day, the sleep-inducing sessions were averaged to obtain a single value (Figure 1).

All comparisons of the percentage of correct responses and spectral power were done using a non-parametric, two-tailed Wilcoxon matched-pair test. The analysis includes the same participants in both the control and the sleep inducing sessions. In the results of this test, lower values mean higher statistically significant levels. The effect size was calculated to determine if the SOP has a greater effect on the components of attention than the sleep deprivation used to induce the SOP.

## RESULTS

In the control session, participants did not fall asleep during the almost 12 minutes of the task; during the sleep-inducing sessions participants had an average sleep latency of 6.72±3.18 minutes.

### Changes in spectral power during the SOP

**Figure 2:**
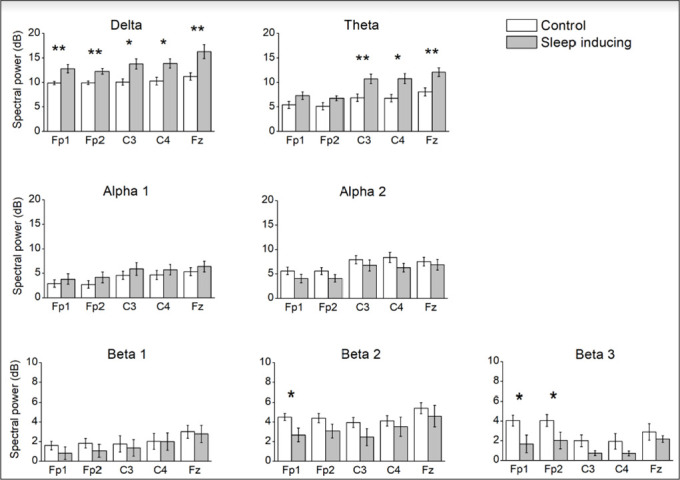
Comparison of the spectral power in delta, theta, alpha 1, alpha 2, beta 1, beta 2, and beta 3 between the last minute of the task in the control session and the last minute before the sleep onset during the sleep-inducing sessions. Bars represent mean ± standard error of the spectral power (dB). *p<0.05, **p<0.01.

When comparing the spectral power of the last minute before the sleep onset in the sleep-inducing session to the power of the last minute of the task in the control session, beta 1 showed no differences but beta 2 showed lower activity in Fp1 (W=3.0, *p*=0.02) and beta 3 had a lower activity in both prefrontal derivations (Fp1: W=3.0, *p*=0.02; Fp2: W=3.00; *p*=0.02) before the sleep onset compared to the end of the control session. Alpha 1 and alpha 2 did not show any differences (Figure 2). Theta activity showed no differences in the frontopolar derivations, although there was a tendency towards significance (Fp1: W=7.0, *p*=0.07; Fp2: W=6.0, *p*=0.05), however it was higher on the central and frontocentral derivations during the last minute before sleep onset, compared to the control (C3: W=1.00, *p*=0.01; C4: W=2.00, *p*=0.02; Fz: W=0.0, *p*=0.01). The activity on the delta band increased on all derivations in the last minute before the sleep onset compared to the control (Fp1: W=0.0, *p*=0.01; Fp2: W=0.0, *p*=0.01; C3: W=4.0, *p*=0.03; C4: W=3.0, *p*=0.02; Fz: W=0.0, *p*=0.01) (Table 1).

**Table 1:** Spectral density for each EEG band during the sleep onset process (SOP).

		Control session	Sleep-inducing session	W	p	d
**Delta**						
	Fp1	9.47 (1.37)	12.20 (2.67)	0.00	0.01	1.62
	Fp2	9.69 (1.29)	12.08 (2.64)	0.00	0.01	1.62
	C3	9.48 (3.00)	13.35 (4.59)	4.00	0.03	1.21
	C4	9.45 (4.49)	13.04 (1.80)	3.00	0.02	1.30
	Fz	10.24 (3.75)	15.08 (2.49)	0.00	0.01	1.62
**Theta**						
	Fp1	4.26 (3.95)	7.11 (1.35)	7.00	0.07	
	Fp2	3.72 (4.56)	6.78 (1.46)	6.00	0.05	
	C3	5.77 (2.86)	9.91 (5.14)	1.00	0.01	1.50
	C4	6.31 (2.87)	10.06 (3.69)	2.00	0.02	1.40
	Fz	6.85 (2.23)	12.52 (4.16)	0.00	0.01	1.62
**Alpha 1**						
	Fp1	2.23 (3.71)	3.02 (6.12)	18.00	0.59	
	Fp2	2.64 (3.52)	2.79 (5.10)	13.00	0.26	
	C3	5.08 (4.00)	4.08 (2.86)	16.00	0.44	
	C4	3.73 (4.59)	4.38 (2.30)	15.00	0.97	
	Fz	6.31 (3.64)	5.31 (3.30)	19.00	0.98	
**Alpha 2**						
	Fp1	4.61 (2.95)	3.74 (1.84)	12.00	0.21	
	Fp2	5.08 (2.15)	3.55 (2.64)	11.00	0.17	
	C3	7.39 (3.75)	6.82 (1.83)	13.00	0.26	
	C4	8.15 (2.99)	5.60 (1.10)	12.00	0.21	
	Fz	7.09 (3.09)	6.00 (2.00)	19.00	0.68	
**Beta 1**						
	Fp1	2.06 (2.00)	1.32 (2.49)	13.00	0.26	
	Fp2	2.06 (1.61)	1.30 (2.27)	17.00	0.51	
	C3	1.14 (2.76)	1.37 (3.64)	19.00	0.68	
	C4	2.49 (2.87)	1.56 (4.86)	19.00	0.68	
	Fz	2.86 (1.75)	2.79 (2.42)	18.00	0.59	
**Beta 2**						
	Fp1	4.55 (0.88)	2.19 (3.07)	3.00	0.02	1.30
	Fp2	4.03 (1.26)	3.15 (2.44)	11.00	0.17	
	C3	3.84 (0.81)	2.36 (1.36)	19.00	0.68	
	C4	4.74 (1.44)	2.91 (2.94)	21.00	0.86	
	Fz	5.32 (0.59)	4.52 (2.00)	13.00	0.26	
**Beta 3**						
	Fp1	4.74 (1.97)	1.29 (2.22)	3.00	0.02	1.30
	Fp2	4.51 (1.30)	2.28 (3.67)	3.00	0.02	1.30
	C3	1.78 (2.62)	-0.02 (5.55)	13.00	0.26	
	C4	2.37 (2.19)	0.76 (3.95)	11.00	0.17	
	Fz	3.89 (2.80)	1.81 (3.16)	13.00	0.26	

**Notes:** Data is the spectral power (dB) of each band compared between the last minute of the control session and the last minute before the sleep onset of the sleep-inducing session. Values are median (interquartile range), the test was a Wilcoxon matched-pair test (*W*), and the effect size was Cohen’s *d.*

### Changes in the components of attention during the SOP

**Figure 3:**
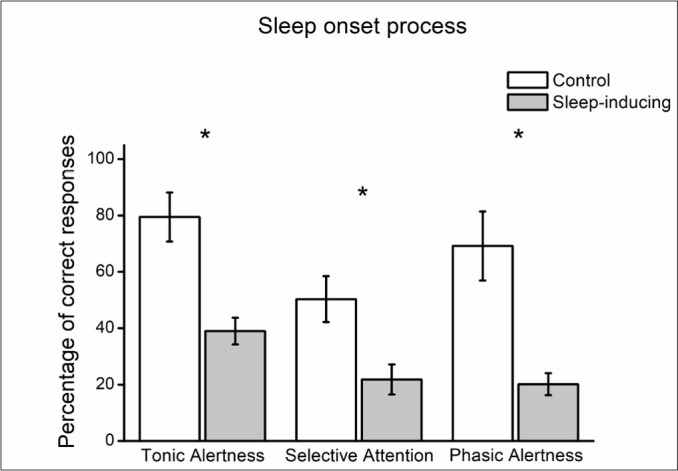
Comparison of correct responses during the last minute of the task in the control session, and the last minute before the sleep onset in the sleep-inducing sessions. Bars represent the mean ± standard error of the mean. *p<0.05.

In average, participants had 40.46% less correct responses in tonic alertness during the last minute before the sleep onset, compared to the last minute of the control task (W=2.00, *p*=0.02). Participants had 28.51% less correct responses in selective attention before the sleep onset, compared to the control session (W=3.00, *p*=0.02). In phasic alertness, participants had 49.03% less correct responses before the sleep onset, compared to the control session (W=1.00, *p*=0.02) (Figure 3). This difference in performance shows that the components of attention are lower during the last minute of the SOP (Table 2). There were no differences in reaction times between the control and sleep-inducing sessions on any of the components of attention.

**Table 2:** Percentage of correct responses for each component of attention during the sleep onset process (SOP) and under sleep deprivation.

Component of attention		SOP (last minute)					Sleep deprivation (first minute)			
	Control session	Sleep-inducing session	*W*	*p*	*d*	Control session	Sleep-inducing session	*W*	*p*	*d*
Tonic alertness	90.00 (32.50)	40.00 (20.85)	2.00	0.02	1.40	100.00 (5.00)	91.25 (16.25)	5.00	0.06	
Selective attention	50.00 (32.75)	25.00 (27.10)	3.00	0.02	1.30	87.50 (17.50)	68.75 (41.67)	3.00	0.04	1.14
Phasic alertness	75.00 (62.50)	25.00 (15.62)	1.00	0.02	1.36	92.50 (25.00)	75.00 (64.58)	5.00	0.13	

**Notes:** Data is the percentage of correct responses compared between the last minute of the control session and the last minute before the sleep onset of the sleep-inducing session, and between the first minute of the control session and the first minute of the sleep-inducing session. Values are median (interquartile range), the test was a Wilcoxon matched-pair test (*W*), and the effect size was Cohen’s *d*.

### Changes in the components of attention under sleep deprivation

**Figure 4:**
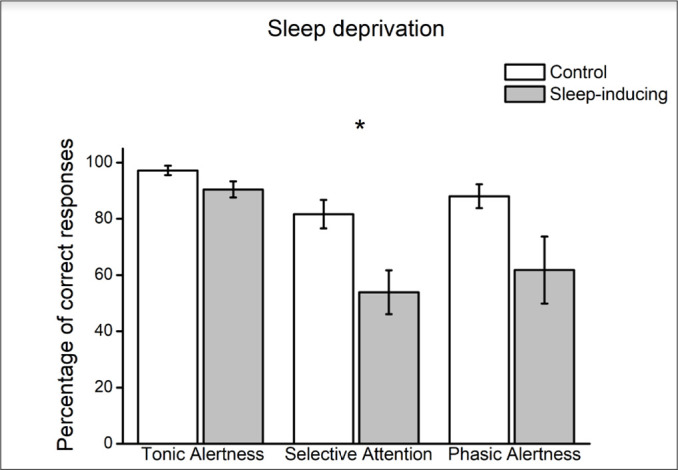
Comparison of the percentage of correct responses during the first minute of the task in the control session, and the first minute of the task in the sleep-inducing sessions. Bars represent the mean ± standard error of the mean. *p<0.05.

In the previous results, it is indistinguishable if the low performance on the components of attention is due to the approaching of the sleep onset or to the sleep deprivation that was used to induce sleep pressure. Therefore, the performance during the first minute of the sleep-inducing session was compared to the first minute of the control session to determine the effect of sleep deprivation. On these comparisons, selective attention was lower by 27.73% at the beginning of the sleep-inducing sessions (W=3.00, *p*=0.04) (Figure 4). The other components did not show significant differences.

## DISCUSSION

Less activity on the fast beta range in frontopolar derivations was accompanied by a decrease in the components of attention, during the last moments of the SOP. In other words, when participants were awake, they had higher prefrontal fast frequency activity and were able to respond to most of the stimuli, when participants were falling asleep they had less prefrontal fast frequency activity and a reduced capacity to respond.

The comparison of spectral power during these two moments showed that before the sleep onset, there is lower activity in the fast beta sub-band, as well as higher activity in the theta and delta bands. The faster beta sub-band (27-34Hz) showed changes in both prefrontal derivations, an area of the cortex that is related to selective and sustained attention^30^, which demonstrates the importance of dividing the beta band during spectral analysis.

These results further confirm that fast activity is related to cognition and they also suggest that fast frequencies are specifically related to the components of attention^9,31,32^. Fast and slow alpha activity did not show changes, as this activity is characteristic of relaxed wakefulness and the two moments being compared are active wakefulness (the end of the control task) and the last moments of the SOP, which are both moments when alpha is expected to be low and not so different.

This study showed an increase in theta in the frontocentral and central derivations during the SOP right before falling asleep, which coincides with the increase other studies have documented in theta over the frontocentral region^33-36^. This increased slow activity has been previously related to a decrease of attentional capacity^12^. Although delta activity usually appears until the beginning of sleep stage 3, it was higher on all analyzed locations before falling asleep, in comparison to a moment where they were not falling asleep. It is important to state that since this particular moment of the SOP includes many events in the 1Hz range (K complexes and vertex waves), this particular frequency was not taken into delta to avoid confusion. The increase in delta activity is independent of these events and, since it seems maximal in the frontocentral derivation, could be of subcortical origin. The appearance of delta activity is a sign that neurons are very synchronized and that they are most likely not responding to external stimuli.

Other studies have documented changes in EEG activity and attention on longer periods before and after the sleep onset. These studies report that right before the sleep onset there is an appearance of slow frequencies (theta and delta), and a reduction of beta activity compared to a state of wakefulness^37^. One of these studies also reports that the capacity to respond to simple stimuli decreases a lot in the minute before sleep onset^38^. Results reported on this study are consistent with those in previous studies.

This decrease in fast EEG activity in the prefrontal areas of the cortex and the increase in slow activity in subcortical regions coincides with the decrease in the components of attention. Performance on the CPT showed that tonic alertness, phasic alertness, and selective attention are low during the last minute of the sleep onset process, compared to a situation with the same stimulation and task conditions but in which the participants were not falling asleep.

A tonic alertness decrease indicates that the capacity to respond to all stimuli in the environment is decreased. Therefore, as the sleep onset approaches, people may be unresponsive to stimuli, which in the case of someone driving a vehicle would largely increase the chances of an accident, as the driver would have difficulties detecting other vehicles or signs on the road.

The phasic alertness component was lower in the last minute of the sleep onset process; this indicates that at this moment people are not benefitting from warning signs that precede stimuli. For example, the horn of another car is in many cases a warning sign of danger, but in this moment of the SOP, it would not improve a driver’s capacity to respond to and avoid that danger.

The low selective attention observed during the last minute of the SOP implies a diminished capacity to give the specific response that is needed when presented with a specific stimulus. For example, a driver that is about to fall asleep is more likely to ignore or to respond inadequately to a traffic signal, hence augmenting the probabilities that a traffic accident occurs. This component was already affected by sleep deprivation, but as the SOP approaches this capacity is reduced even more. Entering the SOP during the day is usually due to sleep deprivation, thus both influences are present at the same time in everyday life.

The findings on tonic alertness coincide with those of Hori et al. (1994)^21^, although the present study found less correct responses during the SOP instead of a lengthening of reaction time. The results of this study also coincide with the observations made by Ogilvie et al. (1991)^13^ and Casagrande et al. (1997)^20^, even though task performance itself was not reported, these studies employed tasks that evaluate attention as a general concept and not as a neuropsychological function with four components. Therefore, the state of the components of attention during the SOP had not been properly analyzed. In contrast, the CPT allowed the independent analysis of three components of attention, showing a greater decrease in selective attention and phasic alertness. This suggests that activities depending on these components will have grave errors if performed during the SOP. This study is, to our knowledge, the first to analyze the spectral power of the EEG signal and the components of attention during the SOP.

In brief, these results show that the fast frequency activity in the prefrontal cortex and slow frequency activity in the central regions is related to a decreased capacity to respond to the environment, to specific stimuli, or after a warning signal even though the person has not yet reached sleep stage 2. It has been reported that during the SOP prefrontal and reticular activity decreases^39,40^. This study confirms there is a decrease in beta activity in prefrontal lobes as well as lower selective attention, a component related to prefrontal activity; similarly, there is an increase in theta and delta activity probably originating subcortically through the influence of the reticular formation^28^, an area that is related to tonic and phasic alertness.

People that enter the SOP due to sleep deprivation while performing important activities, such as learning, driving, operating machinery, and caring for others, are at a high risk of committing a mistake. In order to prevent accidents and perform adequately at work or school, everyone should be aware that their capacity to respond is deficient even before falling asleep.

Even though this study had a small number of participants - and despite the interindividual differences known to characterize the EEG - the differences were strong enough to become significant. Also, since this study was conducted on young people it would be interesting to analyze if other age groups have similar changes in EEG activity and the components of attention during the sleep onset process. During puberty and adolescence, bedtime is delayed^41^ probably due to changes in the light sensitivity of the cerebral mechanisms of sleep and there are also changes in the spectral composition of the EEG signal during wakefulness and sleep. This developmental phase is probably not complete in the age group in which this study and others have studied the SOP.

## CONCLUSION

During the SOP, slow frontocentral EEG activity increases, fast prefrontal EEG activity decreases, and there is a decrease in the components of attention. Young people who sleep less than they need to are prone to enter the SOP during the day even while carrying out an activity, which would result in a lower capacity to perform properly. This means that they are at risk of making mistakes that could lead to serious accidents.
